# Mobile Exergaming in Adolescents’ Everyday Life—Contextual Design of Where, When, with Whom, and How: The SmartLife Case

**DOI:** 10.3390/ijerph15050835

**Published:** 2018-04-24

**Authors:** Ayla Schwarz, Ann DeSmet, Greet Cardon, Sebastien Chastin, Ruben Costa, António Grilo, Josue Ferri, Jorge Domenech, Jeroen Stragier

**Affiliations:** 1 Department of Movement and Sports Sciences, Ghent University, 9000 Ghent, Belgium; ayla.schwarz@ugent.be (A.S.); ann.desmet@ugent.be (A.D.); sebastien.chastin@gcu.ac.uk (S.C.); jeroen.stragier@ugent.be (J.S.); 2 Research Foundation Flanders, 1000 Brussels, Belgium; 3 School of Health and Life Science, Glasgow Caledonian University, Glasgow G4 0BA, UK; 4 Centre of Technology and Systems, UNINOVA, 2829-516 Caparica, Portugal; rddc@uninova.pt; 5 Faculdade de Ciências e Tecnologia da UNL, UNIDEMI, 2829-516 Caparica, Portugal; acbg@fct.unl.pt; 6 Asociación de Investigación de la Industria Textil, AITEX, 03801 Alcoy, Spain; josue.ferri@aitex.es (J.F); jdomenech@aitex.es (J.D.); 7 IMEC-MICT, Department of Communication Sciences, Ghent University, 9000 Ghent, Belgium

**Keywords:** mobile exergame, adolescents, contextual design, everyday life, health intervention

## Abstract

Exergames, more specifically console-based exergames, are generally enjoyed by adolescents and known to increase physical activity. Nevertheless, they have a reduced usage over time and demonstrate little effectiveness over the long term. In order to increase playing time, mobile exergames may increase potential playing time, but need to be engaging and integrated in everyday life. The goal of the present study was to examine the context of gameplay for mobile exergaming in adolescents’ everyday life to inform game design and the integration of gameplay into everyday life. Eight focus groups were conducted with 49 Flemish adolescents (11 to 17 years of age). The focus groups were audiotaped, transcribed, and analyzed by means of thematic analysis via Nvivo 11 software (QSR International Pty Ltd., Victoria, Australia). The adolescents indicated leisure time and travel time to and from school as suitable timeframes for playing a mobile exergame. Outdoor gameplay should be restricted to the personal living environment of adolescents. Besides outdoor locations, the game should also be adaptable to at-home activities. Activities could vary from running outside to fitness exercises inside. Furthermore, the social context of the game was important, e.g., playing in teams or meeting at (virtual) meeting points. Physical activity tracking via smart clothing was identified as a motivator for gameplay. By means of this study, game developers may be better equipped to develop mobile exergames that embed gameplay in adolescents’ everyday life.

## 1. Introduction

Physical activity is known to decrease in adolescence, influenced by the transition from high school to college [1]. Promoting physical activity in adolescence is important, since physical activity habits may track into adulthood [2], although evidence of this is limited [3]. More importantly, physical activity is known to prevent cardiovascular diseases, type 2 diabetes, and cancer in adulthood [2]. Effective strategies to encourage adolescents to engage in voluntary participation in daily physical activity are needed [4,5] among adolescents [6]. Health interventions are increasingly technology-based to reach a large number of people. Such technology-based health interventions are also shown to be effective for behavioral change [7,8,9], including physical activity [10]. The potential of digital games in health promotion interventions was observed in the use of physical activity games [10], also known as exergames. Exergames are any digital games that require more physical exertion or movements than sedentary behavior and include strength, balance, or flexibility activities [11].

Console-based exergames are popular among adolescents [12,13] and considered to be engaging [13,14]. Both commercial “off-the-shelf” exergames (which are primarily developed for entertainment purposes) and serious games (designed to be both educational and entertaining) that are aimed at promoting physical activity were found to be effective in increasing light-to-moderate physical activity [15,16,17]. Experimental studies showed that exergames that are more engaging may lead to greater activity intensity [18,19]. Yet, adolescents tend to play console-based exergames less over time, and research has demonstrated little effectiveness over the long term [13,17]. Integrating an exergame into the everyday life may increase usage of the game and everyday physical activity [20,21].

In comparison to console-based exergames, mobile exergames that can be played on a smartphone increasingly gain acceptance among younger people [22]. Smartphones are strongly integrated in adolescents’ everyday life. In Belgium, the smartphone is the number one indispensable technology device among 15 to 19 years old [23]. Adolescents are known to play digital games [24], and half of the Flemish adolescents play games on their smartphone on a daily basis [25]. This ubiquity of mobile devices as well as the popularity of mobile gaming, suggest that mobile exergames may present an opportunity to increase long-term gameplay by promoting physical activity in congruence with an adolescents’ everyday life and practices [21,26,27,28]. As such, games are no longer restricted to the game’s virtual world, but can be directly integrated into everyday life [29,30]. Gameplay is not restricted to fixed locations [31] and can be done anywhere, including indoor and outdoor locations [22], since mobile exergames increasingly extract accelerometer or pedometer data from smartphones or wearable sensors [19,22,32,33,34,35]. These devices enable real-time activity monitoring [36] and afford immediate or frequent behavioral feedback to the user [37]. In our study, we made use of a T-shirt with an integrated accelerometer (hereafter: smart shirt) paired to a mobile exergame. Smart shirts are suggested to be engaging [38,39], but research on the integration of a smart shirt within gameplay in everyday life is scarce [38]. Mobile exergames and activity sensors provide the opportunity to embed gaming into everyday life, but the context of mobile exergame gameplay needs to be understood to design an engaging and effective solution for physical activity in everyday life, and not just in controlled settings [17,40,41,42,43].

Contextual design is a valid framework to inform game design. It focuses on designing a product from the perspective of how the customer works, by understanding and integrating user’s needs in the design of a new system [44], while also taking the context in which the system will be used into account [40]. In game design, this implies that the user context and non-digital play experiences are considered to look beyond core gameplay mechanics and incorporate a broader approach to the game experience [45]. In order to engage the target users over a longer time, games are, for example, increasingly embedded in the social context of the players [45,46]. Furthermore, contextual factors, e.g., setting and time spent in the virtual world of a game, need to be well aligned with the real world in order to be engaging [47,48].

To date, studies investigating how to integrate mobile exergames in everyday life are scarce [17]. Consequently, there is a need to identify strategies to design games that can be effective when played in everyday life [21]. Formative research with the target group is needed to inform the development phase of a game [49] in addition to the evaluation phase [34]. This study examined the context of gameplay in adolescents’ everyday life to inform game design and the integration of gameplay into everyday life. The study assessed where, when, with whom, and how adolescents’ everyday life can inform mobile exergame design.

## 2. Materials and Methods

This study is part of the European SmartLife project (www.smartlifeproject.eu) that aims to increase physical activity among adolescents, by means of a mobile exergame that is paired with a smart shirt. At the time of this study, it was intended to develop a narrative-driven audio exergame that guides the player through a virtual world. Physical activity, especially lower body movement, in real life is required to proceed in the game. The requirement of physical activity is integrated to the narrative and, instead of providing particular exercises, players are free to decide on how to be physically active. Data from the smart shirt’s sensor adaptively tailor gameplay by providing real-time feedback on the individual’s physical activity intensity. A Bluetooth connection transmits the data from the sensor to the phone and converts into physical activity intensity.

### 2.1. Sample Description and Recruitment

A convenience sample of two secondary schools in Flanders, including eight classes, was used. Both schools offered academic and non-academic track education. Academic track education (e.g., science, language) is a broad form of education preparing students for higher education. Non-academic track education includes technical (e.g., industrial science, administration) or vocational (e.g., woodwork, hairdresser) education. It either offers a general education from a less theoretical perspective and a more technical and practical approach or prepares secondary school students for the labor market immediately after secondary school. In September 2017, a written informed consent form was distributed to the director of the school, to all adolescents of the recruited classes, and to their parents or guardian. Both school principals provided active written informed consent. Adolescents who gave active written informed consent and whose parents or guardian gave passive informed consent were eligible to participate in the study. From the selected classes, three male and three female adolescents were randomly selected (for example every second or third adolescent on the class list).

In total, 49 adolescents, aged 11 to 17 years, with a mean age of 15 years (SD = 1.80), participated in the focus groups. The parents of two adolescents (out of a total of 120 recruited adolescents) did not consent their child to participate in the study (response rate: 98%). None of the adolescents declined to participate. The sample was evenly split by gender and evenly distributed over educational grade. Adolescents in non-academic track education were overrepresented in the sample. Detailed information on the participants is provided in Table 1, and detailed information on the focus groups is provided in Table 2.

### 2.2. Ethical Statement

Ethical approval was received from the Ethics Committee of Ghent University Hospital (project 2017/0254; registration: B670201731695). This study was conducted according to the guidelines laid down in the Declaration of Helsinki. Active written informed consent was obtained from all participating adolescents and school principals, and passive informed consent was obtained from parents of the participating adolescents.

### 2.3. Data Collection

In September 2017, eight semi-structured focus group sessions were conducted during a class hour (50 min). Each focus group consisted of adolescents of the same class, to include adolescents from the same school type, grade, and age in one group. It was expected that using focus groups with adolescents from the same class would foster more interaction [50]. All focus group interviews were led by one moderator, who was familiar with the questioning route and was assisted by a co-moderator and an extra facilitator. The co-moderator asked additional questions and, together with the facilitator, monitored the recording equipment, kept track of time and the interview guide, and organized the logistics.

A semi-structured interview guide was used that contained key questions about the context where the adolescents expected the game could be played, including moments and locations that were suitable to play the game. Furthermore, general introductory questions about current gameplay experiences were asked as warm-up exercises (Table 3).

At the end of the focus group, each participant completed a short questionnaire that asked for socio-demographics, current frequency of gameplay, and physical activity level. Socio-demographic information included gender (female/male), education type (academic/non-academic track education), school grade, and age. A 5-point scale assessed the current frequency of playing digital games and exergames: never, less than monthly, monthly, weekly, several times a week, daily, several times a day. To evaluate the physical activity level of the participants, the Godin Leisure-Time Exercise Questionnaire (GLTEQ) was used to assess the frequency of engaging in mild, moderate, or vigorous exercise in the past seven days, and of engaging in activities long enough to induce sweating [51]. Mild exercise was defined as requiring minimal effort, such as easy walking. Moderate exercise was defined as exercise that is not exhausting, such as fast walking, tennis, or easy swimming. Vigorous exercise was defined as exercise which makes the heart beat rapidly, such as jogging, soccer, or vigorous swimming. On the basis of the Leisure Score Index (LSI) the sum of the metabolic equivalent (MET) values of 3, 5, and 9 METs was used for mild, moderate, and vigorous exercise respectively, multiplied by the frequency of engaging in the activity. GLTEQ is valid for a total physical activity score [52,53]. The cut-point value for moderate-to-vigorous LSI of 24 or higher was classified as active, whereas an LSI of 23 or smaller was classified as insufficiently active.

### 2.4. Data Analysis

All focus groups were audiotaped and transcribed to facilitate the analysis. The data were thematically analyzed by means of Nvivo11 software (QSR International Pty Ltd., Victoria, Australia). The data were coded and analyzed to identify repeated patterns of meaning [54]. An initial coding scheme was created to categorize the responses on the basis of the system-based EU-PAD framework (European-Physical Activity Determinants across the life course) that focuses on physical activity behaviors [55]. In order to create a game that is enjoyable and effective in everyday life, ecological models have demonstrated to be informative [34]. They provide a framework to study the context and may help to inform the design of the exergame. Sub-themes that emerged in the data were added to the coding scheme. All facilitators (n = 3) were trained to code the data for thematic analysis with the final coding scheme. The data set was independently coded by three researchers and compared until consensus was reached. The interrater reliability was found to be substantial (Kappa = 0.73), suggesting adequate agreement between the coders [56].

## 3. Results

Repeated patterns were grouped into themes that can further inform the contextual design of the mobile exergame in everyday life. Five main themes were identified: (1) physical activity; (2) meaningful locations; (3) suitable timeframes; (4) social context; (5) activity tracking via smart clothing.

### 3.1. Gameplay and Physical Activity

The survey data showed that the adolescents played passive digital games more frequently (e.g., several times a day, daily, or several times a week) than exergames (e.g., monthly, less than monthly, or never). Only a minority of adolescents played exergames at least weekly (Table 4). The sample largely reported frequently engaging in moderate-to-vigorous physical activity (n = 43), compared to a small group who was insufficiently active (n = 5) (missing n = 1). Most participants (often n = 30; sometimes n = 19) engaged in activities long enough to work up sweat and were member of a sports club (n = 30).

Qualitative findings indicated that the adolescents preferred to have a broad choice in the types of physical activity in the game: “…that you can vary a bit, everything a little bit, if you have been running for one month, that you can choose a different sport in the next month.” (Focus Group (FG 6)). They wanted these physical activities to be alternated with moments of rest and other game challenges. Ideas on specific physical activities during gameplay can be summarized to:

- Physical activities that adolescents are already performing (e.g., football, biking, running, dancing, yoga);
- Activities that they have identified in earlier exergames (e.g., boxing, bowling);
- Running activities that can be linked to different game settings;
- Challenges and jumping activities that relate to free running and obstacle courses.

### 3.2. Meaningful Locations

The adolescents imagined they would play an exergame indoor as well as outdoor. The place where they wanted to play the game depended on external conditions (e.g., the weather) and internal conditions such as their physiological state (e.g., being tired). For example, when it was raining or when they felt tired they would prefer playing the game indoors, at home. Outdoor locations were restricted to their own neighborhood or close-by area, because of practical as well as safety reasons. The adolescents identified cities and parks as appropriate places to walk and run: “You can make an obstacle course somewhere on a terrain, where you have a lot of barriers you need to pass”, (FG 4).

Outdoor locations were often desired in combination with a virtual map that recommends new places: “…we have places where you can do fitness exercises nearby. Maybe you can have a map, showing where to find these spots and indicating the difficulty of the exercises…”, (FG 2). “Or that people can upload the information themselves, like I walked there and I can recommend this place”, (FG 7). The adolescents also wished to have a virtual map that facilitates social gatherings: “…I see that Adrian is close-by to where I am and then I can send him a challenge to do together”, (FG 8). Being inside or being outside enabled different types of movements, ranging from stretching, dancing, and fitness inside to running and jumping outside.

### 3.3. Suitable Timeframes for Gameplay

The adolescents indicated time availability to be an important influencer on whether they played games and whether they were physically active. One challenge named to participate in voluntary physical activity was the capability of combining school (hours) with leisure time sports. The adolescents often felt tired after school of “sitting all day at school”, (FG 8). Many adolescents tended to immediately sit down on the couch, as soon as they got home. The adolescents were particularly motivated to play games to fight boredom (e.g., to play mini-games while sitting in the bus), relax, or think of something else. Yet, they missed a motivator to be physically active after school.

The adolescents indicated certain times they were available that would be an opportunity to play an exergame:

- Just after school;
- After finishing school work;
- Commuting to school;
- After having dinner;
- During the weekend;
- During holidays.

Many adolescents commute to school by bike or have to walk to the train station. Others take the bus and, on their bus ride, often play games with mini challenges. Moments while waiting for or sitting on the bus or train, or while biking or walking outside were considered possible times to play the game. The adolescents liked the idea that active commuting could be counted as active gameplay: “Otherwise you are only sitting in the bus or train, then you are only sitting, probably listening to music.” (FG 1). However, others expressed that the distance to school would be too far for them to switch from train or bus to bike.

The idea of playing the game during school breaks received mixed reactions, since school policies often do not permit mobile phone usage during school hours, and other adolescents reported to prefer interacting with friends instead of playing games: “…it is not social for those who are not playing. In case we are playing together, it is again not sociable towards others who are not playing along. In any case, it is not sociable.” (FG 4).

Playing the game before going to bed was identified as a barrier, since adolescents stated they would not engage in physical activity before bedtime. To fit gameplay within the moment they found suitable, adolescents indicated they would like to be able to choose from particular challenges that required different completion times, e.g., 5 min, 10 min, 15 min. “…that you can choose, like, ah that one only takes 5 min, so I can easily do that one, or that one takes 20 min or even longer.” (FG 8).

### 3.4. Social Context

The adolescents indicated to be often influenced by their peers or family to play a game. They tend to download a game that their friends or family members are already playing: “I am just playing this because my mum plays this as well, and then we want to beat each other.” (FG 5). This facilitated comparison to other players, e.g., in rankings. “Some games also have rankings that are linked to Facebook, and then you can see who of your Facebook friends is first.” (FG 8).

The adolescents frequently use various social media applications on their mobile phones, especially to connect with friends. Their preferences for playing a game, either in competition or in cooperation with other players, are strongly linked to this: “…people who do a lot of sports probably want a game that is competitive, but other people like this less.” (FG 8). They want to be able to play in teams and be able to compare themselves to others, e.g., in rankings or leaderboards.

Physical activity was often reported to occur in a social context. When the adolescents are not engaging in organized sports, they would organize physical activity privately and meet with friends, e.g., to go running. Integrating the game in the social context could further facilitate gameplay.

### 3.5. Activity Tracking via Smart Clothing

The adolescents found the sensing capability of smart clothing an interesting tool to track their physical activity. They were especially interested to assess the time and intensity they put into a particular exercise and compare it to their friends’: “To know for example how many calories you have burned or how intensively you have been active is a motivator”, (FG 8). Furthermore, they felt that the smart clothing could also give insights in personal physical activity intensity and reward them for their efforts. The smart clothing could serve as a trigger to start being physically active: “For example, when you are sitting at home and you are in your daily clothes, you are less motivated to exercise. I think if you have a shirt in the game, then you are more motivated, that is, “look, I am wearing the shirt and I will leave right away”, (FG 8).

The opportunity to customize their own clothing would increase their willingness to wear it and their motivation to play the game. Participants said they would like to be able to customize the T-shirt with their (team) name, their game character, a brand, or the logo of the game. Also, they would like to adapt the shape (e.g., long sleeve), color, and material (e.g., regular shirt or sport shirt). They wished to include a flag on the shoulder or a badge that allowed the player to level up in the game and simultaneously upgrade the individual shirt. Shirt customization could both reflect individual customization as well customization to identify their team identity or achievements. This showed that smart clothing needs to be further integrated in the social context of the adolescents.

The adolescents provided mixed feedback with regard to wearing the shirt during gameplay. Some adolescents would wear it throughout the day, whereas others would only wear the shirt when they are actually playing the game: “I would even wear it without playing the game” (FG 4). Some female adolescents preferred the development of a top, instead of a T-shirt, they could wear underneath everyday clothes. “It needs to be a top or something similar, something that you can wear underneath” (FG 2).

## 4. Discussion

This study examined how a mobile exergame for adolescents can be optimally designed to fit in adolescents’ everyday life. Results revealed that outdoor gameplay should be restricted to the personal living environment of adolescents. At-home activities should be available for indoor gameplay when outdoor gameplay is not possible. The adolescents assumed that various timeframes in their everyday life offered opportunities to play a mobile exergame. The social context of the game was considered a key motivators for playing the game. By considering where, when, with whom, and how, an engaging exergame can be built that adapts throughout the gameplay [57]. As such, challenges of integrating mobile exergames and smart clothing in everyday life [17,38,41,42,43] can be addressed. In the following paragraphs, we discuss (1) where, (2) when, (3) with whom, and 4) how adolescents’ everyday life can inform mobile exergame design, on the basis of the results of this study.

The results indicated that both indoor and outdoor locations were acceptable for adolescents to play a mobile exergame. They preferred outdoor locations to be restricted to their own neighborhood, but the game could also suggest new, safe locations outside of their immediate surroundings. Several games are location-based or integrate GPS data [22,27,32,33,58] and consider the current location of the player. Most studies on exergames are implemented in school settings [17,32,34] and only focus on one location. Considering multiple locations that are visited daily by adolescents is novel to our study and may inform gameplay. A retrospective research on the commercial mobile exergame ‘Pokémon go’ revealed that gameplay took mainly place in urban areas and neighborhoods with a small number of minority groups. In addition, the players identified risks when playing the game, such as visiting unknown or dangerous places, or risks that are caused by the player’s movement while looking at the smartphone screen [59]. This emphasizes the importance to embed the gameplay in the personal environment of the player, for example in his or her neighborhood, or adapt it to known locations, and only suggest safe locations. Furthermore, ‘Pokémon go’ focusses on physical activity outdoors and does not promote indoor physical activity [60], as console-based exergames do [17]. In comparison, our results showed that the adolescents desired to be active at both indoor and outdoor locations. To our knowledge, no earlier research exists on how locations can be considered in exergame development. Considering suitable locations in the development phase that are accessible to adolescents and are suitable for a mobile exergame is assumed to facilitate gameplay in everyday life [27,61]. It is advised that players have the opportunity to change locations during gameplay. More importantly, they should be able to choose between indoor and outdoor locations and for individual preferences. After informing the game design, further research is needed to assess locations where adolescents actually play the mobile exergame [62,63].

The adolescents in our study indicated that gameplay prior to bedtime was not a suitable timeframe. Research shows that gameplay before bedtime influences sleep quality [64,65] and is accordingly advised to be excluded from game designs. Before or after-school hours, in addition to commuting to school, were considered suitable timeframes for playing a mobile exergame. Research reveals that after-school hours are a critical period of physical activity and sedentary behavior among adolescents. Adolescents however often engage in technology-based activities at that time [66]. The results indicated that moments when the adolescents are already engaging in technology-based activities to overcome boredom may be replaced with the active play of a mobile exergame. In addition, research shows that transportation stops or stations were often used by adolescents to wait for friends or public transportation [67]. Our study adds that during these timeframes, including waiting time and after-school hours, a mobile exergame may be interesting to play. Furthermore, the results indicated that gameplay should not interfere with school hours or school breaks. Research implemented in schools indicated that ecological factors such as school policy (e.g., smartphone permission during school breaks) may interfere with actual gameplay [61]. However, when implemented in the class curriculum, games show promising effects on increasing physical activity [17]. Pupils tend to choose voluntarily to play a game, instead of participating in class, as this kind of activity is a new and out-of-the-ordinary way of learning [42]. In comparison, study effects during leisure time, where exergames have to compete with other leisure activities or games and require self-regulation of the adolescent, are less consistent [17,42,43]. Our findings showed that gameplay should be adaptable to how much time a person has available. Game design can be further informed by assuming that, during leisure time, suitable timeframes exist when a mobile exergame would be interesting to play. In order to identify individual timeframes during leisure time, smartphone data may help. The notion of detecting human activity through smartphones is increasingly implemented in science [68]. The adoption of such techniques could be used in order to analyze and infer different timeframes during the day in which there is a high probability of leisure time and boredom. The detection of such situations can trigger suggestions to adolescents for playing an exergame. Such techniques would require collecting and processing data from each individual user to identify patterns in human leisure time with the support of machine learning algorithms. Further research is required to investigate the acceptance among the target group towards adaptable tailoring, with regard to triggering notifications during assumed suitable timeframes.

Our findings reinforce the need to integrate the social context in the design of a game. Social integration has been applied in many exergames for adolescents [19,32,33,69]. Research shows the importance of social integration in gameplay [21,22,70,71,72,73,74,75,76], for example that, compared to the single-player mode, the multiplayer mode can achieve greater energy expenditure and increased heart rate [73]. In the literature, especially cooperative activities are identified as a strategy that may promote usage of and adherence to the exergame [77]. Our findings showed that the adolescents wish to play the game together with their friends or family. Furthermore, playing in teams, in competition, or cooperation was highly valued by the adolescents. In addition, offering the multiplayer mode was especially appreciated when being physically active outdoor (e.g., physically meeting friends) or indoor (e.g., virtually comparing to friends). Incorporating these strategies into gameplay is therefore hypothesized to increase usage of and adherence to the exergame. Further research is needed to assess whether setting individual preferences for social player mode increases the effects and engagement of exergame gameplay [78].

The adolescents in our study considered activity tracking via smart clothing to be a potential motivator for playing the game [38]. While wearable activity tracking has been applied in previous studies [19,22,34], our research presents specific ideas with regard to the use of smart clothing for adolescents. Especially, the option to customize and personalize a smart shirt seems to be needed to motivate wearing the shirt both during and outside of the gameplay. In smart clothing research, a shift is noticed from research involving professional athletes to targeting the general population [79]. Besides the wish of adolescents to track their physical activity data, previous studies considered that accelerometers are useful tools to monitor physical activity in adolescents [5,80,81]. In addition, accelerometers not only provide objective measurements that ensure the effectiveness of the physical activity performed, but also provide reliable information about patterns and real-time data [82]. To ensure the accuracy of the results and player’s comfort, the sensor placement needs to be determined in the design phase. Placing the sensors close to the body helps to avoid false measurement values, as evident from previous research [83,84,85].

Overall, it appears that our results regarding where, when, with whom, and how to play an exergame are closely related to each other. For example, adolescents wanted to have a virtual map (where) that facilitates social gathering (with whom). The smart clothing (how) may track individual and group data that enables adaptable tailoring, e.g., triggering gameplay during suitable timeframes (when), collecting rewards (how), and comparing results with other players (with whom). The smart clothing not only offers the possibility to enable gameplay and individual tailoring but is also hypothesized to contribute as a motivator to gameplay. The possibility of customizing smart clothing was highly valued by our research participants and may not only connect to gameplay, but also connect smart clothing to the social context. Serious mobile exergames show to include social features [28,34] and increasingly embed learning systems to enable tailored feedback [33]. Increasingly, games are designed that take the context and habits of the player into account, get to know the player during gameplay, and, accordingly, provide tailored and persuasive messages and action [86,87]. Commercial mobile exergames show to be engaging [88] but mainly focus on outdoor physical activity. Results from a study in a related domain of smartphone intervention, showed that location-based maps are convenient to promote social interaction, to provide new route recommendations, and to manage friendships [89]. Our study adds that next to the outdoor locations, it may be important to include indoor locations and consequently adapt them to suitable activities. Activity tracking not only seems important for the gameplay itself but also can serve as an additional motivator for playing the game, via customizable smart clothing. Furthermore, we assume that a game with adaptive tailoring is better suited to tailor the gameplay to the specific context of the player. By doing so, gameplay can be better embedded in adolescents’ everyday life. By means of this study, game developers and data analysts may be better equipped to develop mobile exergames that can be integrated in the everyday life of adolescents and that are individually adaptable.

### Strengths and Limitations

The generalizability of our study findings is influenced by the limitation that the sample consisted of adolescents of only one country, in particular Flemish adolescents. This may not be generalizable to everyday life practices of adolescents in other cultural contexts. Our sample mainly consisted of physically active adolescents. This share was higher than in the general Flemish population [90]. Possibly, they may have been more interested in an exergame than less physically active adolescents might have been. The results of this study should therefore be considered with care regarding generalizability. The study also has several strengths. It contributes to the limited research on the topic of mobile exergames in everyday life. Furthermore, it used a diverse sample as regards age, gender, and education type for qualitative analyses. Our sample shows an overrepresentation of adolescents of non-academic education. Exergame research often has an underrepresentation of adolescents of non-academic education, and the purposeful oversampling in our study takes into account the views of this often underrepresented group of adolescents. By means of contextual design, the different needs of the target group could be taken into account. Furthermore, the independent coding of three researchers contributed to the reliability of the study findings [91].

## 5. Conclusions

The location of gameplay should be restricted to the personal living environment of known places but should also provide opportunities for both outdoor and indoor activities at home and vary from running outside to fitness exercises inside. Leisure time and travel time to and from school as well as after-school hours are suitable timeframes for playing a mobile exergame. Integrating exergame gameplay in specific timeframes, for example when adolescents are playing minigames out of boredom on public transportation, may influence the active travel time. The social context of the game is important and may consist of adolescents playing in teams or finding each other at (virtual) meeting points. Activity tracking via smart clothing may connect the where, when, with whom, and how and serve as an additional motivator for gameplay. An exergame that can be integrated in daily activities, played with adolescents’ peers, and connected to smart clothing appears a promising concept design. These findings may support game developers targeting adolescents in creating suitable persuasive mobile exergames well embedded in adolescents’ everyday life.

## Figures and Tables

**Table 1:** Characteristics of participating adolescents.

Characteristic	n = 49
**Gender**	
Female	22
Male	27
**Age**	
<12 years old	1
12–14 years old	18
15–16 years old	20
17–18 years old	10
**Grade**	
First grade	12
Second grade	19
Third grade	18
**Type of Education**	
Academic track education	13
Non-academic track education	36

**Table 2:** Characteristics of the focus groups.

Characteristic	FG ¹	FG	FG	FG	FG	FG	FG	FG
	1	2	3	4	5	6	7	8
**Gender**	(n = 6)	(n = 6)	(n = 6)	(n = 6)	(n = 6)	(n = 6)	(n = 7)	(n = 6)
Female	3	2	0	3	6	1	3	4
Male	3	4	6	3	0	5	4	2
**Age**	16–17	14–16	11–13	15–16	12–13	16–17	14–15	16–17
**Type of Education**	Non-academic	Non-academic	Non-academic	Non-academic	Non-academic	Non-academic	Academic	Academic

^1^ FG = Focus group.

**Table 3:** Semi-structured focus group guide for adolescents.

Theme	Key Question	Supporting Questions
Digital games	A. In your opinion, what does a perfect digital mobile game look like that motivates you to play?	1. Are you playing any digital, mobile, or exergames?
Active digital games	B. What should a mobile game look like to make you want to move more?	2. Which activities would you like to do in the game?
Active gaming in everyday life	C. How could the mobile exergame fit into your everyday life?	3. When would you like to play the game? 4. With whom would you like to play the game? 5. How much time would you spend playing the game?
Smart shirt	D. What do you think about wearing a T-shirt that measures your activity to play the game?	6. When would you wear the shirt? 7. How would you like the possibility to individually customize your shirt? 8. When you see and feel this shirt, what would you change so that it would suit your preferences?

**Table 4:** Characteristics of the gameplay frequency.

	Digital Games (n = 49)	Exergame (n = 49)
Never	4	17
Less than monthly	6	14
Monthly	6	10
Weekly	9	5
Several times a week	14	3
Daily	8	0
Several times a day	2	0
